# Status Epilepticus After an Exploratory Ingestion of Cannabis Edibles

**DOI:** 10.7759/cureus.91463

**Published:** 2025-09-02

**Authors:** Kevin Rivera, Alek Q Adkins, Brian P Murray, Hannah Hays

**Affiliations:** 1 Emergency Medicine, The Ohio State University Wexner Medical Center, Columbus, USA; 2 Medical Toxicology, Nationwide Children’s Hospital, Columbus, USA; 3 Emergency Medicine, Wright State University, Dayton, USA

**Keywords:** cannabis, pediatric, status epilepticus, tetrahydrocannabinol, toxicology

## Abstract

An 18-month-old previously healthy male presented unresponsive with generalized tonic-clonic activity. Seizures recurred despite multiple benzodiazepine doses, requiring intubation and mechanical ventilation. Extensive workup, including CT, MRI, lumbar puncture, respiratory panel, and blood cultures, failed to find an alternative cause for status epilepticus. Toxicologic testing confirmed a urine 11-nor-9-carboxy-tetrahydrocannabinol (THC-COOH) concentration >500 ng/mL and serum THC-COOH concentration of 256 ng/mL. Urine benzoylecgonine was also present at 74 ng/mL; no serum cocaine concentration was obtained. The patient returned to his neurologic baseline and was discharged after five days. To our knowledge, this is the first reported case of status epilepticus associated with laboratory-confirmed cannabis exposure in a toddler. Clinicians should consider THC toxicity in pediatric patients presenting with refractory seizures. Early recognition may guide acute management, targeted toxicologic testing, and child safety interventions.

## Introduction

Pediatric cannabis exposures have increased markedly over the past decade, following the legalization of medical and recreational cannabis in many states and the growing popularity of cannabis edibles. At higher exposures, delta-9-tetrahydrocannabinol (Δ9-THC) can paradoxically increase excitability through preferential suppression of GABAergic transmission, providing a mechanistic basis for proconvulsant effects. Recognition of this possibility is particularly important in the emergency department, where cannabis toxicity may not be an immediately obvious cause of refractory seizures. A retrospective study utilizing National Poison Data System (NPDS) data showed an increase in reported annual cannabis exposures from 207 in 2017 to 3,054 in 2021. During that time, reported pediatric cannabis exposures per 1,000 NPDS pediatric cases rose from 0.2 in 2017 to 3.6 in 2021 [1-3].

Toddlers are particularly vulnerable, with reported effects including lethargy, ataxia, and respiratory depression [4]. A retrospective chart review at a tertiary academic center showed age-dependent, statistically significant differences in these clinical outcomes [4]. Case series have reported seizure-like activity following pediatric cannabis exposures [5]. However, all of these cases involved single short-duration, self-limited seizures; there are no known case reports describing status epilepticus as a clinical effect. Cannabis exposure, particularly at high milligrams per kilogram (mg/kg) in toddlers, can lead to seizures and not just sedation or somnolence.

We describe an 18-month-old male who developed status epilepticus following exploratory ingestion of cannabis edibles. Confirmation of exposure was established through detection of 11-nor-9-carboxy-THC (THC-COOH), the principal metabolite of THC and a reliable biomarker of cannabis ingestion. This case emphasizes the need for a broad differential diagnosis in pediatric seizures and draws attention to the potential proconvulsant effects of cannabis in overdose.

## Case presentation

A previously healthy 18-month-old 12.7 kg male, born at 37 weeks, presented to a community emergency department (ED) for unresponsiveness with generalized seizure-like activity witnessed by family members. Initial vital signs were blood pressure (BP) 100/61 mmHg, heart rate (HR) 136 bpm, respiratory rate (RR) 30 rpm, oxygen saturation (SpO₂) 97% on room air, and a core temperature of 33.7 °C. A finger-stick glucose reading was 111 mg/dL. No evidence of trauma, infection, or drowning was identified on initial evaluation. On questioning, the family acknowledged the presence of cannabis edibles in the home. There was no history of prior child protective services involvement for abuse, nutrition, or supervision concerns. He was externally rewarmed and received 1 mg intranasal midazolam, resolving seizure activity in less than one minute, but leaving persistent encephalopathy. He remained encephalopathic without return to baseline when a second tonic-clonic episode occurred approximately 45 minutes later, prompting administration of IV lorazepam (0.1 mg/kg) and warmed saline. The exact semiology of each seizure was not documented in nursing or provider notes. Due to persistent depressed mental status and a venous blood gas (VBG) showing a pCO₂ of 68 mmHg (reference range 35-45 mmHg), he was emergently intubated using intravenous etomidate 3.8 mg and succinylcholine 20 mg. No complications such as emesis or airway trauma were documented.

Empiric intravenous antimicrobials were initiated, including ceftriaxone 1,270 mg once daily for two days, vancomycin (initially 190.5 mg then increased to 280 mg every six hours for two days), and acyclovir 190 mg once. Initial laboratory evaluation (Table 1), lumbar puncture (Table 2), and radiographic studies, including a chest X-ray and computed tomography (CT) of the head (Table 3), were unremarkable.

**Table 1:** Initial serum laboratory diagnostics.

Test	Result	Reference range
White blood cells	13.4 thou cells/mm3	6.0 - 17.0 thou cells/mm3
Hemoglobin	11.8 mg/dL	10.5 - 14.5 mg/dL
Platelets	371 thou cells/mm3	130 - 400 thou cells/mm3
Sodium	137 mEq/L	135 - 145 mEq/L
Potassium	3.5 mEq/L	3.5 - 5.2 mEq/L
Chloride	103 mEq/L	98 - 111 mEq/L
Calcium	9.4 mg/dL	8.5 - 10.5 mg/dL
CO2	21 mEq/L	23 - 33 mEq/L
Blood urea nitrogen (BUN)	23 mg/dL	7 - 22 mg/dL
Creatinine	<0.2 mg/dL	0.4 - 1.2 mg/dL
Glucose	125 mg/dL	70 - 108 mg/dL
Osmolality	279 mOsmol/kg	275 - 300 mOsmol/kg
Lactic acid	2.2 mmol/L	0.5 - 1.9 mmol/L
Procalcitonin	0.04 ng/mL	0.01 - 0.09 ng/mL
Aspartate aminotransferase	37 U/L	5 - 40 U/L
Alanine aminotransferase	23 U/L	11 - 66 U/L
Alkaline phosphatase	301 U/L	30 - 400 U/L
Total protein	6.2 g/dL	6.1 - 8.0 g/dL
Albumin	4.4 g/dL	3.5 - 5.1 g/dL
Total bilirubin	0.2 mg/dL	0.3 - 1.2 mg/dL

**Table 2:** Lumbar puncture cerebrospinal fluid analysis.

Test	Result	Reference range
Appearance	Clear colorless fluid	N/A
Nucleated cells	0/cumm	0 - 20/cumm
Red blood cells	28/cumm	0/cumm
Protein	22 mg/dL	12 - 60 mg/dI
Glucose	95 mg/dL	40 - 80 mg/dL

**Table 3:** Initial radiologic diagnostics.

Imaging Modality	Results
Chest X-ray	No acute abnormalities. No incidental findings. Normal study.
CT head	No acute abnormalities. No incidental findings. Normal study.

**Figure 1:**
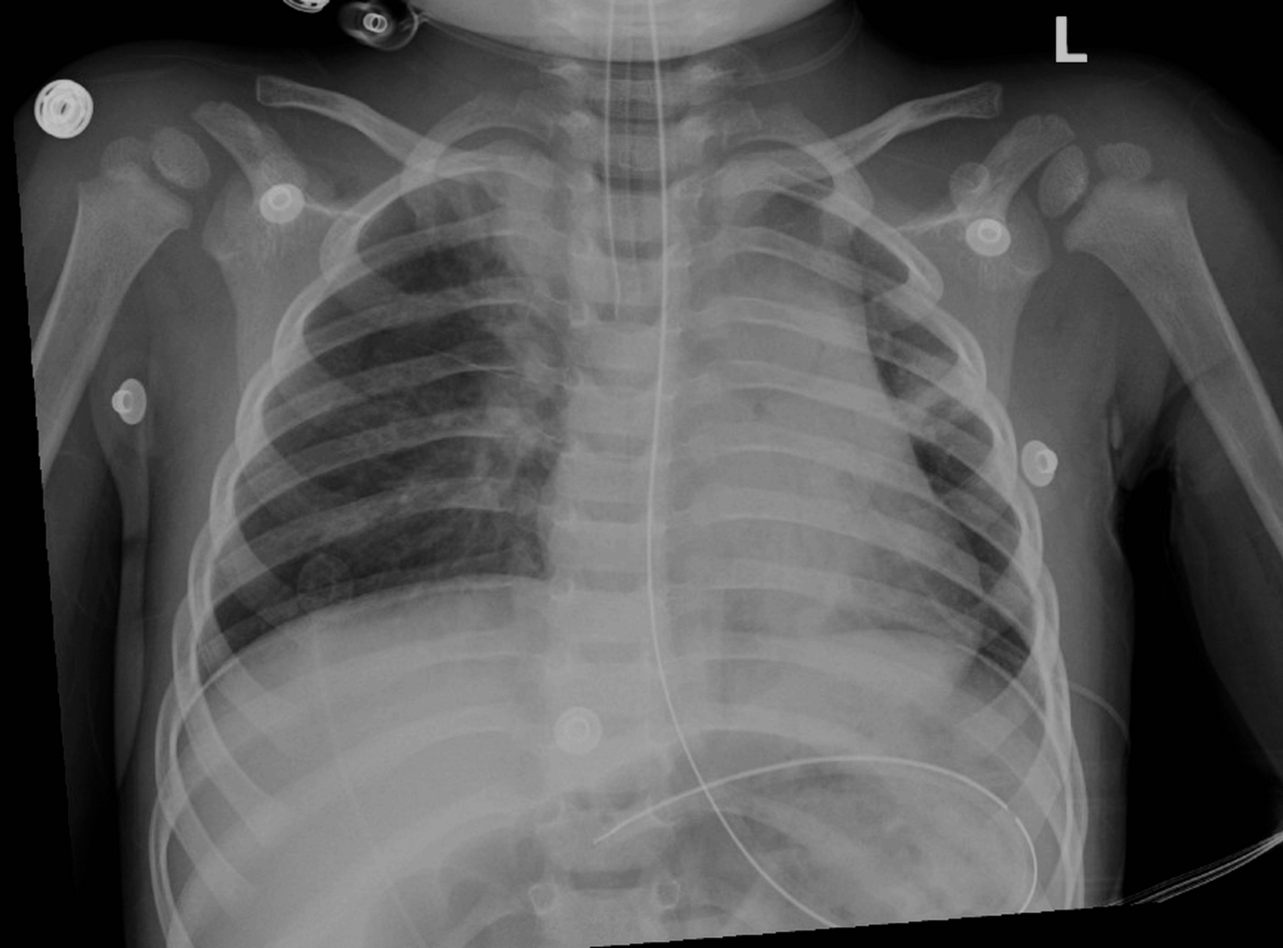
Chest radiograph obtained after intubation demonstrating no acute cardiopulmonary abnormalities.

The normal chest radiograph is shown in Figure 1. A respiratory viral panel (nasal PCR) was negative. The patient was transferred to a regional quaternary pediatric hospital.

**Figure 2:**
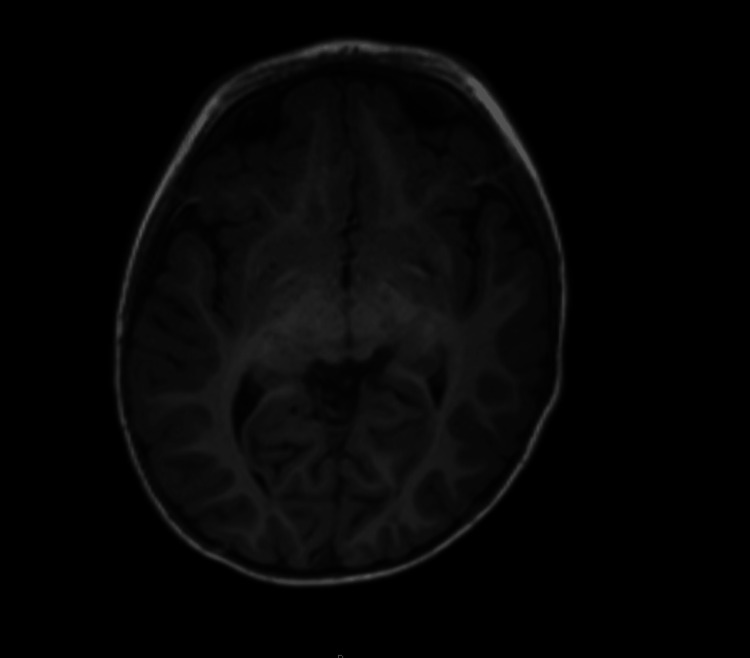
Axial T1-weighted magnetic resonance imaging (MRI) of the brain demonstrating no acute infarction, mass effect, hemorrhage, or structural abnormalities. The temporal lobes are unremarkable.

**Figure 3:**
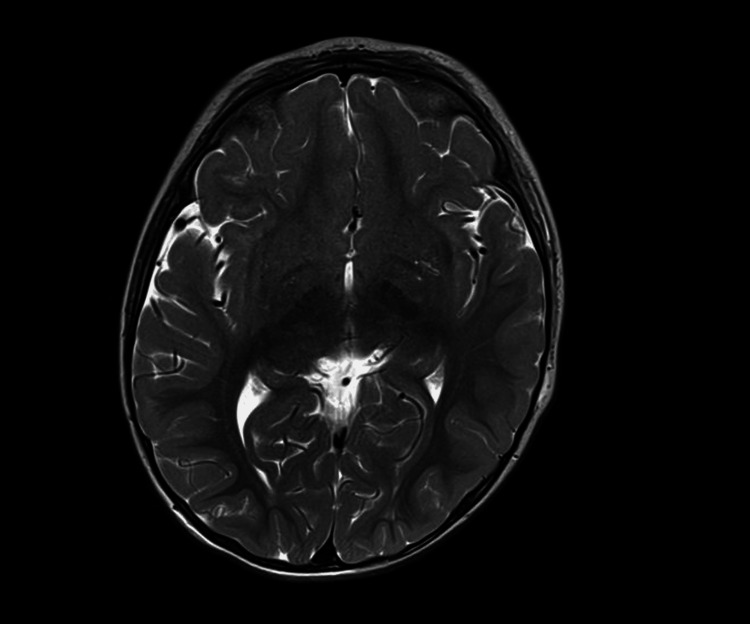
Axial T2-weighted MRI of the brain showing no edema, encephalomalacia, or abnormal signal intensity. No structural or temporal lobe abnormalities were identified.

On arrival at the quaternary center, he was receiving continuous midazolam at 0.1 mg/kg/hr, fentanyl 1 microgram/kg/hr (mcg/kg/hr), and maintenance fluids of D5W in 0.9% saline at 48 mL/hr. Neurology confirmed status epilepticus based on recurrent tonic-clonic activity requiring continuous benzodiazepine infusion rather than progression to long-term antiepileptic therapy, consistent with a presumed toxicologic etiology. A routine urine drug screen revealed 11-nor-9-carboxy-THC (THC-COOH) >500 ng/mL. A cocaine metabolite, benzoylecgonine, was detected above the lower assay threshold but below the reporting cutoff. Confirmatory liquid chromatography-mass spectrometry (LC/MS) performed at an external laboratory returned a benzoylecgonine concentration of 74 ng/mL (reference cutoff: ≥50 ng/mL considered positive). A repeat urine immunoassay four hours later was negative. Serum cocaine level was not obtained. Blood cultures showed no growth through day 5. Magnetic resonance imaging (MRI) of the brain, including both T1- and T2-weighted sequences with temporal lobe cuts, demonstrated no acute or structural abnormalities (Figures 2, 3). An electroencephalogram (EEG) performed during hospitalization showed no epileptiform activity and was otherwise normal. Social work and child protection services were engaged. The patient’s mother confirmed cannabis edibles in the home. The confirmatory LC/MS result required 10 days as a send-out test, limiting its immediate utility for acute management. No long-term antiepileptic agents were administered, as seizures were attributed to toxicologic exposure. Child Protective Services (CPS) was contacted.

On hospital days three and four, intermittent tremors were observed, primarily in the left upper extremity but occasionally involving both arms and the jaw. Neurology noted that the movements appeared to be stimulus-induced rather than intention tremors. These episodes resolved spontaneously by hospital day five and were not associated with EEG abnormalities. After extubation, the patient was noted to be sleepy but arousable, with normal tone, no focal neurological deficits, and intact strength across extremities. At discharge, tremors had resolved, and he was neurologically intact.

## Discussion

This appears to be the first reported case of status epilepticus most plausibly driven by tetrahydrocannabinol (THC) ingestion in a pediatric patient. Pediatric patients are more susceptible to severe toxic effects due to smaller body mass, immature hepatic metabolism, and differing neurodevelopmental vulnerability [1,6,7]. In this case, the patient experienced persistent encephalopathy between seizures. Transient post-extubation tremors, primarily involving the upper extremities and jaw, may have reflected residual sedation, postictal activity, or persistent neuroexcitation from THC. These movements resolved spontaneously and were not associated with epileptiform EEG findings or MRI abnormalities, further supporting a nonstructural and reversible etiology. Because a toxicologic etiology was suspected, the care team prioritized benzodiazepines and supportive care over long-term antiepileptic drug loading. Although more potent synthetic cannabinoids such as Δ9-THCP have been described in the literature, these were not suspected given the parental report of conventional THC edibles in the home and absence of other exposure history. While the precise dose and source could not be confirmed, toxicologic analysis revealed a serum THC-COOH concentration of 256 ng/mL, far exceeding levels seen with recreational use [8]. This finding provides strong objective evidence of significant exposure.

THC exerts its effects as a partial agonist at cannabinoid type 1 (CB1) receptors, which are densely expressed in the cortex, hippocampus, and cerebellum. While low doses may suppress excitatory neurotransmission and appear anticonvulsant, high concentrations preferentially inhibit GABAergic signaling, leading to network disinhibition and increased seizure susceptibility. This biphasic profile has been demonstrated in both animal models and pediatric case series, where THC exposure was linked to heightened hippocampal excitability and reduced seizure threshold, particularly in immature neurons [6]. In a scoping review, cannabis exposure was associated with increased seizure incidence in 10 of 11 studies involving pediatric and general populations [7]. These findings suggest delta-9-THC may have proconvulsant properties in overdose.

THC is primarily metabolized by cytochrome P450 enzymes CYP2C9, CYP2C19, and CYP3A4 [9]. These enzymes exhibit variable expression and activity in early childhood, leading to increased susceptibility to either accumulation or toxic metabolite production. Such variability may help explain the spectrum of pediatric presentations from mild sedation to status epilepticus.

Other causes of status epilepticus were reasonably excluded. Normal CSF protein and glucose, absence of nucleated cells, and negative cultures made bacterial and viral meningoencephalitis unlikely. Normal electrolytes, calcium, and glucose excluded metabolic precipitants. Normal EKG excluded dysrhythmias. Normal cerebral imaging excluded intracranial hemorrhage, developmental malformations, encephalitis, or brain malignancy. Negative blood cultures excluded bacteremia. Although benzoylecgonine was detected at 74 ng/mL by confirmatory LC/MS, the initial immunoassay signal was below the reporting threshold and a repeat screen four hours later was negative, raising doubt about the clinical significance of this finding. While seizures themselves can be considered part of the sympathomimetic toxidrome, this patient did not demonstrate persistent or escalating features such as hypertension, diaphoresis, mydriasis, or tachyarrhythmia that would typically support cocaine as a primary cause [10]. Combined with the absence of sustained sympathomimetic findings and the prolonged clinical course, these results make cocaine a less likely primary driver. However, because benzoylecgonine was confirmed on LC/MS and false positives are rare, co-exposure cannot be completely excluded. This interpretation is supported by the parental report that cannabis edibles were present in the home at the time of ingestion, providing a clear exposure source. 

Limitations include absent detailed seizure semiology and uncertainty regarding the THC dose or source of the edibles. Nonetheless, toxicologic confirmation with markedly elevated serum and urine THC-COOH supports substantial exposure. This case highlights the need for clinicians to include cannabis exposure in the differential diagnosis of new-onset pediatric seizures, even in the absence of a witnessed ingestion or known THC source.

## Conclusions

To our knowledge, this is the first reported case of status epilepticus most plausibly driven by tetrahydrocannabinol (THC) ingestion in a pediatric patient. While THC appears to be the precipitating agent, co-exposures cannot be fully excluded. Although benzoylecgonine was detected, the absence of sustained sympathomimetic features and a negative repeat test made cocaine less likely as the primary cause. The lack of prior documentation of this association makes recognition particularly difficult, as clinicians may not readily attribute prolonged seizure activity to cannabis toxicity. This case demonstrates that high-dose THC exposure in toddlers can provoke severe, sustained neuroexcitation requiring aggressive airway and seizure management. Without timely intervention, such presentations carry a high risk of hypoxic injury, long-term neurologic sequelae, or death.

Confirming THC exposure through targeted toxicologic testing and ruling out alternative causes underscore the need to keep cannabis on the differential for pediatric seizures. In this case, confirmatory liquid chromatography-mass spectrometry results required several days for return, underscoring a practical limitation for many clinicians. Because turnaround times can exceed the acute management window, emergency physicians often must act on presumptive findings and clinical judgment rather than definitive toxicology. Notably, seizure control relied primarily on benzodiazepines and supportive toxicology management rather than escalation to long-term antiepileptic drugs (AEDs), reflecting the presumed toxicologic etiology. As cannabis products become increasingly available, prevention through public health education, safe storage, and policy measures will be essential to reduce the risk of potentially fatal exposures.
